# Mussel Shells, a Valuable Calcium Resource for the Pharmaceutical Industry

**DOI:** 10.3390/md20010025

**Published:** 2021-12-24

**Authors:** Magdalena Mititelu, Gabriela Stanciu, Doina Drăgănescu, Ana Corina Ioniță, Sorinel Marius Neacșu, Mihaela Dinu, Raluca-Ioana Stefan-van Staden, Elena Moroșan

**Affiliations:** 1 Clinical Laboratory, Food Hygiene Department, Faculty of Pharmacy, Carol Davila University of Medicine and Pharmacy, Traian Vuia Street, 6, 020956 Bucharest, Romania; magdalena.mititelu@umfcd.ro (M.M.); corina.ionita@umfcd.ro (A.C.I.); elena.morosan@umfcd.ro (E.M.); 2 Department of Chemistry and Chemical Engineering, Ovidius University of Constanta, 124 Mamaia Blvd., 900527 Constanta, Romania; 3 Pharmaceutical and Computer Physics Department, Faculty of Pharmacy, Carol Davila University of Medicine and Pharmacy, Traian Vuia Street, 6, 020956 Bucharest, Romania; doina.draganescu@umfcd.ro; 4 Proffesional Farma Line, 116 Republicii Street, 105200 Baicoi, Romania; neacsu_23@yahoo.es; 5 Pharmaceutical Botany and Cell Biology Department, Faculty of Pharmacy, Carol Davila University of Medicine and Pharmacy, Traian Vuia Street, 6, 020956 Bucharest, Romania; mihaela.dinu@umfcd.ro; 6 Laboratory of Electrochemistry and Condensed Matter, National Institute of Research for Electrochemistry and Condensed Matter, Splaiul Independentei Street, 202, 060021 Bucharest, Romania

**Keywords:** mussel shells, calcium salts, calcium levulinate, acute toxicity

## Abstract

(1) Background: The mussel (*Mytilus edulis*, *Mytilus galloprovincialis*) is the most widespread lamellibranch mollusk, being fished on all coasts of the European seas. Mussels are also widely grown in Japan, China, and Spain, especially for food purposes. This paper shows an original technique for mussel shell processing for preparation of calcium salts, such as calcium levulinate. This process involves synthesis of calcium levulinate by treatment of *Mytilus galloprovincialis* shells with levulinic acid. The advantage of mussel shell utilization results in more straightforward qualitative composition. Thus, the weight of the mineral component lies with calcium carbonate, which can be used for extraction of pharmaceutical preparations. (2) Methods: Shell powder was first deproteinized by calcination, then the mineral part was treated with levulinic acid. The problem of shells generally resulting from the industrialization of marine molluscs creates enough shortcomings, if one only mentions storage and handling. One of the solutions proposed by us is the capitalization of calcium from shells in the pharmaceutical industry. (3) Results: The toxicity of calcium levulinate synthesized from the mussel shells was evaluated by the method known in the scientific literature as the Constantinescu phytobiological method (using wheat kernels, *Triticum vulgare* Mill). Acute toxicity of calcium levulinate was evaluated; the experiments showed the low toxicity of calcium levulinate. (4) Conclusion: The experimental results highlighted calcium as the predominant element in the composition of mussel shells, which strengthens the argument of capitalizing the shells as an important natural source of calcium.

## 1. Introduction

Fish and seafood are an excellent source of easily digestible protein, vitamins, minerals, and essential fatty acids. Obviously, it is very important that natural products come from low pollution environments, in order to avoid contamination with different pollutants [1,2]. Mussels from the Romanian Black Sea coast are recognized as abundant marine organisms that are easily accessible and with valuable therapeutic and nutritional potential. The mussel lives in colonies, perching on rocks in the water and forming banks. The viability of mussels is assessed by their achievement. If they are alive, the valves close immediately. Mussels feed on phytoplankton and organic particles by filtering water. Numerous research studies have drawn attention to the risk of accumulation of toxic substances in high concentrations for marine organisms due to the pollution of the marine environment. Filter organisms are among the most affected in the category, which includes mussels. Consumption of contaminated marine products can cause serious illness and even lead to death. For this reason, the quality of the marine environment from which the products intended for consumption come is very important [3,4,5,6]. Mussel meat is a source of high-quality protein and also abundant in phosphatides, which have a beneficial effect on liver activity. Additionally, mussel meat contains a large number of trace elements, including copper, cobalt, manganese, and zinc, together with amino acids and vitamins (PP, B1, B6, B2, E, and D). First of all, mussel meat is a very rich source of quality digestible proteins. In general, due to its beneficial properties, this meat can replace the meat of domestic animals [7,8,9,10]. It has been established that lipids in mussels contain prostaglandins (PG) and their precursors, polyunsaturated fatty acids (AGPN). Thus, polyunsaturated fatty acids, such as linolenic and arachidonic, represent 30–40% of total fatty acids, and such an amount is rarely found in animal fats. These compounds are valuable due to their immuno-modulatory, antioxidant, hypocholesterolemic, and anti-inflammatory action. Analyses with gas–liquid chromatography and mass spectrometry showed that in the composition of lipids in mussels, 37 types of fatty acids were found, of which more than 6% are unsaturated, and the total polyun-saturated fatty acids of linoleic, linolenic, eicosatetrenoic, eicosapentenoic, docosahexaenoic type represents around 28–29% [11,12]. The presence of PG-A prostaglandins was also observed, as well as PG-E compounds with a positive effect on the cardiovascular system. According to clinical studies, a balanced diet rich in omega-3 fatty acids improves bowel health, which could have benefits in the direction of obesity and type 2 diabetes [13,14,15]. In terms of mineral content, the researchers found more than 30 macro- and microelements in mussel meat; the ability to assimilate microelements in mussel meat is favored by the fact that the vast majority of them are related to organic substances [7,8,10].

The shells represent the major part in terms of weight (56–61%). The major component of the shell composition is calcium carbonate (around 94%) [16,17]. The issue of shells generally resulting from industrial processing of marine shellfish is given due importance in countries with the “marine economy”. Such wastes representing up to 80% of the processed raw material are reason enough for concern, if only storage and manipulation were mentioned. One recovery solution would be their calcination for calcium oxide, but the amount of energy required greatly reduces profitability. An alternative may be use as fodder flour, but side effects have occurred in this respect as well, related to digestion problems and intestinal inflammation in animals. A method for recovery of calcium in crustacean shells is provided in the literature, involving calcium chloride obtained by acid attack of crushed shells, deproteinized by boiling in a 1% solution NaOH, which is more complex; however, it requires prior filtration for removal of the protein part and thorough cleaning of the mineral for elimination of traces of alkaline minerals to avoid additional acid use. In the context of increasing consumption of fish and seafood worldwide due to awareness of the need for a healthy diet, rich in valuable nutrients to keep the body in optimal condition, the problem of capitalizing mollusc shells as natural sources of calcium, among others for the pharmaceutical industry, becomes a viable one with multiple advantages: economic efficiency, and capitalization of waste with substantial benefits on environmental conservation and on the health of the population [18]. 

The aim of this paper is to propose a new secondary raw material to prepare pharmaceutically pure calcium salt: calcium levulinate. 

## 2. Materials and Methods

### 2.1. Extraction of Calcium Levulinate from Mussel Shell Waste

For the recovery of mussel shell calcium, an original technology has been designed involving conversion of calcium carbonate, the major component of shell composition (about 94%) [19,20]. The technology comprises two main steps: shell deproteination for removal of the organic component; treatment of the mineral component with various acids to obtain calcium salts useful in the pharmaceutical industry.

The purpose of the proposed technology for mussel shell calcium recovery is to provide more efficient means for industrial mussel processing for extraction of active ingredients, applied on either waste resulting from the food industry or on actual flesh, also in line with current technological trends for environmental protection [21]. 

The process proposed for recovery of mussel shell calcium was adapted from Peniston’s process [19] for industrial use of crustacean shells, and is made up of the following sequence of operations:

1. The raw material is brought to powder to increase the contact area and thus the effectiveness of reagents used;
2. The purpose of the treatment by boiling in 1% alkaline KOH solution is to release the organic component;
3. The mineral cake (the major component) has to be rinsed under pH control to remove alkali traces, which, otherwise, in numbers, would contaminate the yields (calcium salts) and increase use of acid;
4. Processes for the preparation of the various salts share the step of acid attack in a reaction vessel. This was designed in such a way as to allow gradual addition of the acid (by the funnel mounted on upper, tapped side), to enable thorough use of calcium carbonate in the shells and elimination of the resulting reaction CO_2_ from the system. Connection to a vacuum pump hastened the exhaust, also securing a certain level of foam;
5. In particular, certain purification and recrystallisation means were applied for each type of salt.

Utilization of calcium levulinate was introduced by the American Pharmacopoeia as a solution for injection in calcium and allergy therapy [22]. Gordon et al. [23] reported that calcium levulinate solution for injection is not irritating for intravenous administration as compared to other calcium salts; in addition, it has great stability and does not crystallize over time, whereas its calcium content exceeds that of calcium gluconate by 40%. One method for calcium levulinate preparation has been described by Proskouriakoff in 1933, [24] consisting of treatment of a 20% levulinic acid solution at 90 °C with calcium carbonate brought to portions. The solution is further concentrated by evaporation over a water bath, until it is reduced to half the volume, and then cooled to complete crystallization of the calcium levulinate [24]. 

**Figure 1:**
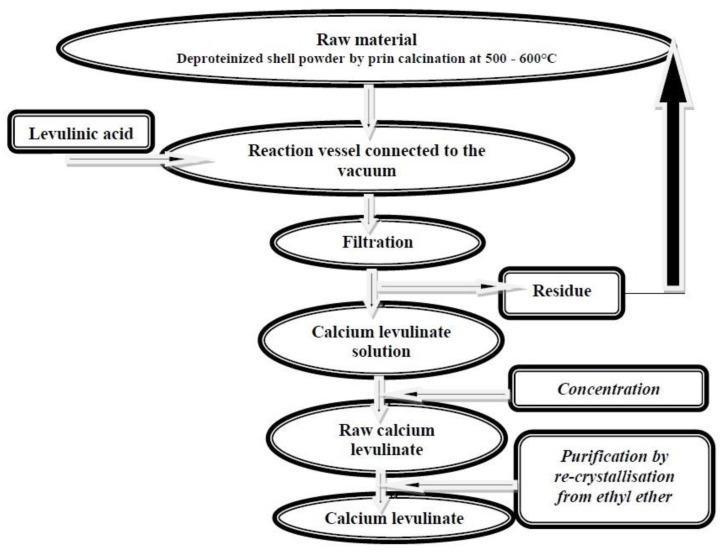
Flowchart for calcium levulinate technological extraction from mussel shell.

Mihele and Mititelu [19] prepared the calcium levulinate from mussel shells by treatment of *Mytilus galloprovincialis* shells with levulinic acid (Figure 1).

The main advantages of the original method for calcium levulinate preparation are: (i) the capitalization of mussel shells through a simple and efficient method, given the abundance of the raw material; (ii) extraction of a calcium organic compound, calcium levulinate, that is readily absorbed and well-tolerated, and used in medical practice internationally even if not mentioned in any edition of the Romanian Pharmacopoeia.

After harvesting, mussels were rinsed and shells were separated from the flesh. Out of 2.50 kg whole mussels, 0.30 ± 0.05 kg mussel flesh and 1.20 ± 0.20 kg shells were obtained. Rinsed and dried shells were powdered and deproteinized by calcination. The powder was calcinated at 500–600 °C for 30 min to 1 h and the mineral sample obtained from deproteination was treated with 98% levulinic acid solution in a reaction vessel under suction.

Particle sizes of the shell powder were obtained by the sieving and sorting method, using a CISA Sieve Shaker Mod. RP 10, produced by Cisa Cedaceria Industrial, Spain. The powder was passed under mechanical shaking through a set of sieves with well-known mesh sizes, and placed under each other in ascending order of a finesse degree.

The particles had sizes in the 80–600 μm range, with the highest amount, about 60%, being between 160 and 315 μm. 

Basically, a small excess shell powder was used to ensure full use of levulinic acid. Treatment with levulinic acid solution was based on the stoichiometric calculation for a CaCO_3_ amount estimated at around 93% in shells; deproteination was considered able to remove the bulk of the organic substance. The calcium levulinate was turned into solution and the unreacted mineral part separated by filtration and recycled. The calcium levulinate resulting solution was then evaporated in the drying oven at 105–110 °C and the crude calcium levulinate obtained was purified by re-crystallization from absolute ethyl alcohol. The yield of the salt relative to the levulinic acid consumed was 91.5%. Thus, from 387 g levulinic acid (3.33 mol) and 250 g shell powder, we obtained 410.84 g of calcium levulinate (1.52 mol).

The reaction was carried out at room temperature and the reaction time was 90 min. An increase in reaction temperature could shorten the duration and increase the efficiency of the process, according to other similar studies regarding obtaining calcium levulinate from the capitalization of egg shells [25]. 

For lack of a specific monograph in the Romanian Pharmacopoeia, the calcium levulinate thus prepared was analyzed and characterized according to provisions of the American Pharmacopoeia [22]. The melting point (122.5 °C) indicated the salt of calcium levulinate anhydrous (with melting temperature 123 °C) and also the characteristics of salt obtained (white to pale yellow powder).

The purified salt (calcium levulinate anhydrous) was also analyzed by IR spectroscopy using a Nicolet 6700 FT-IR spectrometer; in addition, the UV spectrum was obtained of a 1% calcium levulinate solution prepared from shells, using a CAMSPEC M330 UV-VIS spectrometer. 

Quantitative elemental analysis was expressed as a percentage of the quantitative ra-tios between the atoms of the studied organic compound. A Perkin-Elmer 2400 Series II CHNS/O was used. The samples were weighed in tin micro-cans using a Perkin Elmer mi-crobalance. The combustion temperature was set at 850 °C, and in the reduction zone, the temperature was 500 °C. Cystine was used as an analytical standard. For each analyzed sample, two determinations were performed, using different amounts from the sample (~1.5 mg and ~2.5 mg). The result was calculated as the average of the two determinations.

### 2.2. Calcium Levulinate Anhydrous Toxicity Evaluation

#### 2.2.1. Acute Toxicity Evaluation of Calcium Levulinate on Plant Cells

Caryopsis’s embryonic roots of wheat (*Triticum vulgare* Mill. Variety Dropia 2005 ICCPT from Fundulea) treated, germinated under laboratory conditions was used as biological material. The principle of the method is to determine the highest dilution of the active solution which, depending on the duration of action, and the film, influences karyokinetic root elongation. The wheat test method devised by Prof. Constantinescu [26] is convenient because the wheat kernels immediately respond to the action of the substances, thereby allowing a short testing time (4–5 days), while ensuring the accuracy and reproducibility of the results. Additionally, diploid karyotype is 2n = 42 chromosomes, where this number of chromosomes allows to easily see all mitotic alteration films.

For the test, we used a solution of calcium levulinate (10.00%) synthesized and purified from salts of mussel shells. From the 10.00% calcium levulinate solution, the following test solutions were obtained by dilution: 6.66% solution, 5.00%, 3.33%, 0.66%, 0.06% (Table 1).

Wheat kernels, selected to be homogeneous, were soaked with water for 24 h at room temperature (18–25 °C), then placed to germinate in Petri dishes with a diameter of 15–25 cm on the substrate filter paper wetted with water. When the main roots reached a length of 1 cm by 10, the germinated wheat kernels were inserted in a Petri dish containing 15 mL of test solution. Root elongation was measured at the same hour for 5 days. The test was repeated to check the reproducibility of results. Observations were made regarding the morphological changes, such as appearance and the number of rootlets, appearance, and epicotyl length in order to study the microscopic resected embryonic stem wheat, specifically in the meristematic root key at a distance of about 5 mm from the top and stained (with slight heating) with orcein diluted acetic dye with high affinity to chromatin in an acidic environment (the acid pH is required for the hydrolysis of the chromatic material of the meristem, which then turns red-purple). Orcein is a natural dye extracted from lichen genera *Lecanora* and *Rocella*. Stained preparations were then examined by immersion in cedar oil using a Nikon Labophot 2 optical microscope (Ob. 10× and Ob. 100×) digital camera. These macroscopic observations supplemented by microscopic examination revealed the effect of the tested on root elongation and development of shoots from explants of the *Triticum vulgare* mill; and the influence on the rate and conduct of the test solutions mitosis in root meristematic cells of *Triticum vulgare* mill. Triplicate determinations were performed for each sample. Results are expressed as mean ± SD (standard deviation) of triplicate analysis.

#### 2.2.2. Acute Toxicity of Calcium Levulinate Evaluation on Animals

For acute toxicity testing, Swiss albino mice (males, 5 weeks old, and 18–20 g) were used. Animals were housed in groups of ten in a temperature-controlled room (20 ± 1 °C) with a 12 h light–dark cycle. They were allowed access to food and water ad libitum. All animal manipulations were approved by the Scientific Research Ethics Commission of Carol Davila University of Medicine and Pharmacy established under the Animal Protection (code of ethical conduct 2145/12/02/2020) Animal Welfare Act 1999 [27,28]. 

Three doses were administered to the mice under the arithmetic progression of calcium levulinate. The substance was dissolved in sterilized distilled water and the aqueous solutions were administered i.p. in groups of 10 in the mouse laboratory. After each dose, the lethality was determined within batches, and motor events were followed up for the animals [29,30,31].

Statistical analysis of the experimental results was performed using Origin 6.0 software for statistical calculation, resulting in the median lethal dose (LD50), the regression equation, and correlation coefficient for *p* < 0.05. The experimental results representing the mortality batches placed in the experiment, the dose administered, recorded at 24 h, the values of the logarithm of the corresponding dose batches to which were no survivors, and mortality recorded at 24 h in the same groups were introduced into the calculation program, Origin 6.0.

The recorded effects (lethality in the batch) are represented in the ordinate, the abscissa, and the administered doses. For the transformation of exponential dose–response curve in a straight line, we used a logarithmic scale (log10) on the abscissa. The obtained relationship, called regression, characterizes the functional connection between the administered doses, and the measured effect was expressed as: y = a + b × logx, where y = ordinate, the obtained effect; x = abscissa, the administered dose; a = constant or originally ordered; b = inclination straight line (tangent of the angle which is formed by the front right abscissa). The sign of b indicates the direction of the link. The value of b shows changes (increases or decreases) and the average variable y (effect) shows an increase of a variable log x (log dose).

To measure the relationship (link) between the two variables, the administered dose and the lethal effect, the following indicators were used for correlation: the correlation coefficient, and the coefficient of determination.

The expression of the relationship between the administered dose and lethality is the Pearson correlation coefficient, “R”. The coefficient can range from −1 to +1. When R tends to zero, the connection is weaker; when R tends to 1 (plus or minus), the link is more intense and stronger; in other words, in this case, a small change in dose will result in a change to the determined effect.

The coefficient of determination R^2^ is the square of the correlation coefficient and measures the linear dependence between the studied two variables and can range between 0 and 1. It is usually expressed as a percentage indicating how much of the variation is explained by the variation dose effect. 

#### 2.2.3. Analysis of Mineral Composition of Mussel Shells 

**Figure 2:**
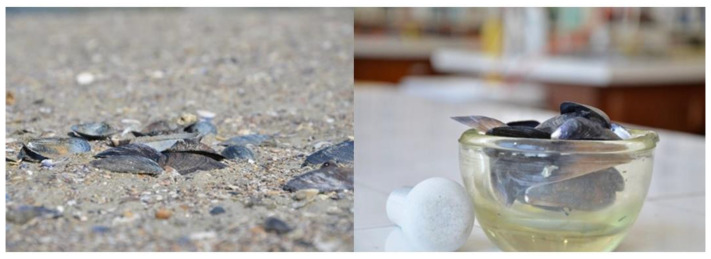
Mussel shells.

Mussel shells were harvested from the Romanian Black Sea coast (Figure 2).

Mussel shells were rinsed with warm distilled water several times; then, the shells were dried in a conventional oven for three days at 50 °C, and ground. 

Macro-elements: Calcium (Ca), Phosphorous (P), Magnesium (Mg), and Sodium (Na), micro-elements: Iron (Fe), Zinc (Zn), Manganese (Mn), Chromium (Cr), Lead (Pb), Cadmium (Cd), and Mercury (Hg) content in mussel shells were determined according to the Association of Official Analytical Chemists (AOAC) [32,33,34]. All these measurements were done in triplicate. For the analysis of the elements, 0.5 g of the shell sample was weighed and digested in screw-cap bottles with concentrated high-purity nitric acid; bottles were heated for 6 h and opened several times to release CO_2_ build-up; and digested samples were diluted to 100 mL using distilled water. The solutions were then shaken and filtered through no. 2 filter paper. The solutions obtained were further diluted to 1:100 prior to the analysis with Inductively Coupled Plasma Mass Spectrometry (ICP-MS) using DRC-E Perkin Elmer equipment, in triplicate. All reagents used were of analytical grade (Merck). 

Statistical analysis of the collected data was performed using open-source software (R Core Team 2019). Statistical significance was accepted for alpha-level 0.05. Because some samples from our study were too small for a classical approach, we applied a robust ANOVA version for comparing our datasets [35].

## 3. Results and Discussion

### 3.1. Characterization of Calcium Levulinate

**Figure 3:**
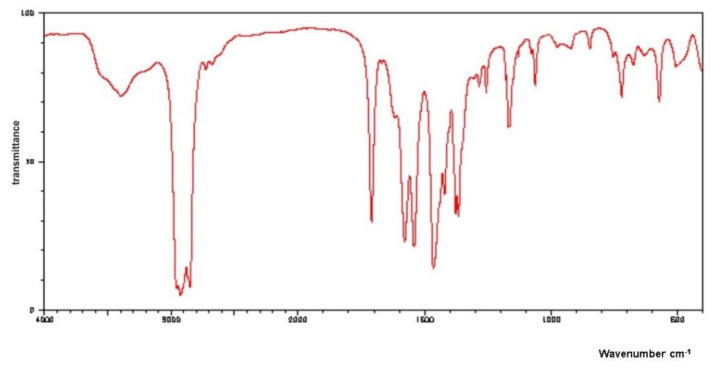
IR spectrum of the calcium levulinate prepared from mussel shells.

The efficiency of the calcium levulinate preparation process in relation to levulinic acid use was 91.5%. The IR spectrum of the obtained calcium levulinate (Figure 3) showed a vibration band characteristic for the C=O group at 1706.4 cm^−1^, a band of the vibration characteristic for the group COO^−^ group at 1566 cm^−1^, as well as a vibrational band characteristic to the OH enol group at 3383–3453.9 cm^−1^ [36]. 

**Figure 4:**
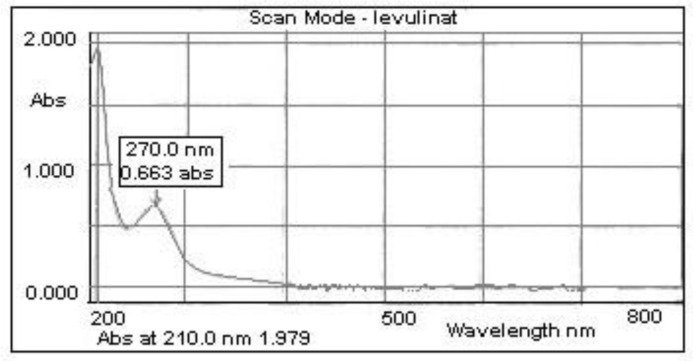
UV spectrum of the calcium levulinate obtained from mussel shells.

The UV spectrum of calcium levulinate obtained from mussel shells is shown in Figure 4.

The characteristics of calcium levulinate prepared from mussel shells are shown in Table 2, and it can be observed that the calcium levulinate thus obtained meets the requirements of the pharmaceutical industry; therefore, it can be used as a starting material for the preparation of medicinal products [22,36].

The results of the elemental analysis are presented in Table 3.

Based on the carbon content of the sample of 44.04 ± 0.6% and that reported to the expected theoretical content of 44.43% (in calcium levulinate anhydrous), the content of calcium levulinate anhydrous in the sample was calculated as 99.12 ± 1.18%. The presence of very small nitrogen and sulfur content in the sample indicates the possibility of the existence of other salts from the impurities of the raw material. 

### 3.2. Calcium Levulinate Toxicity Evaluation

#### 3.2.1. Macroscopic Examination

*Triticum* root length increased compared to the control sample for each solution. The average values for each sample tested for root elongation, within five days and using the technique of linear measurements, are given in Table 4.

E% = Effect% = [A (1 − 6) − Control sample / Control sample] × 100

**Figure 5:**
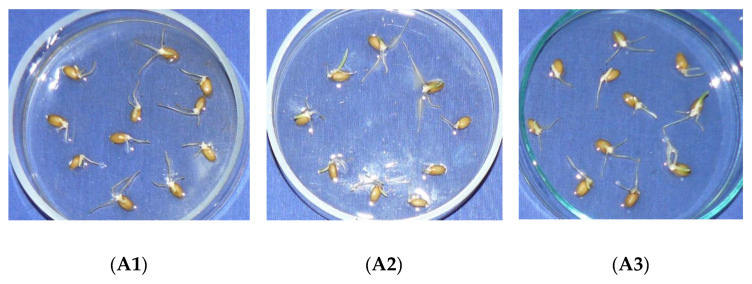

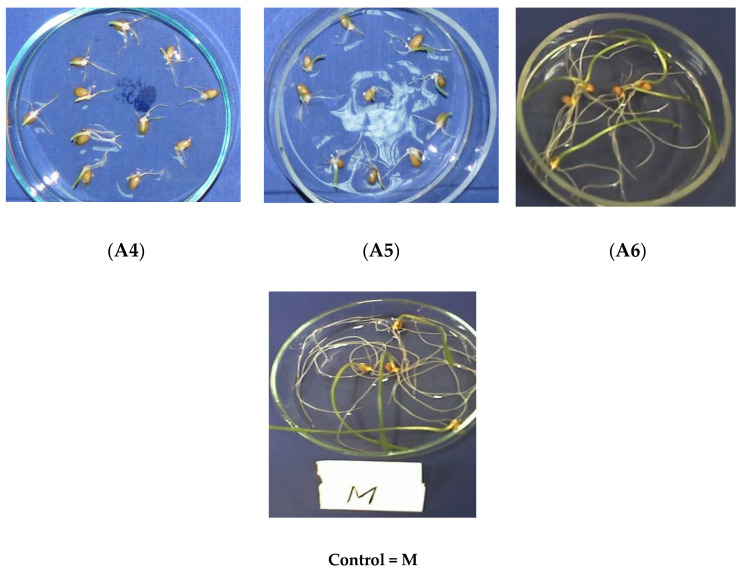
Photos showing the effects of the solutions of calcium levulinate on root elongation; where A = test solutions of calcium levulinate with different concentrations: (**A1**)—10.00% calcium levulinate solution, (**A2**)—6.66% calcium levulinate solution, (**A3**)—5.00% calcium levulinate solution, (**A4**)—3.33% calcium levulinate solution, (**A5**)—0.66% calcium levulinate solution, (**A6**)—0.06% calcium levulinate solution; Control M-blank solution.

The results of the macroscopic observations of the fifth day are shown in Figure 5.

The analysis of the data presented on the root elongation *Triticum vulgare* embryo produced by aqueous solutions under study shows that: in the case of the calcium levulinate solutions at high levels (10 g%, 6.66 g%, 5 g%, 3.33 g%, 0.66 g%), inhibition of root elongation occurs by over 80% compared to the control, which indicates a very strong inhibitory effect; in the case of lower concentrations of the active substance (0.06%) taken in the work, it was found that the root elongation calcium levulinate inhibition compared to the control is produced at a rate of 17.94%, which means that the calcium levulinate does not induce clear inhibition at this concentration; instead, calcium lactate inhibits root elongation by 50.52% [26].

#### 3.2.2. Microscopic Examination

**Figure 6:**
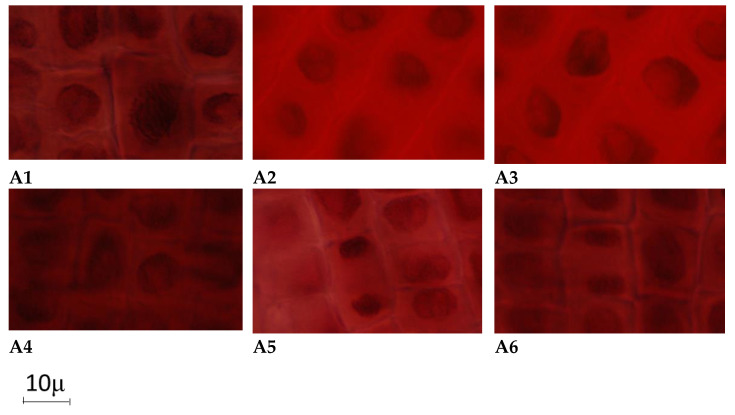
Photomicrographs showing the effect on cell division phases of the solutions of A-calcium levulinate, (**A1**)—precipitated nuclear material, (**A2**)—cell wall corrugated, (**A3**)—cytokinesis inhibition and hypertrophied nucleus, (**A4**)—metaphase disorganized hypertrophied nucleolus, (**A5**)—telophase bridged, (**A6**)—elongated nucleus.

**Figure 7:**
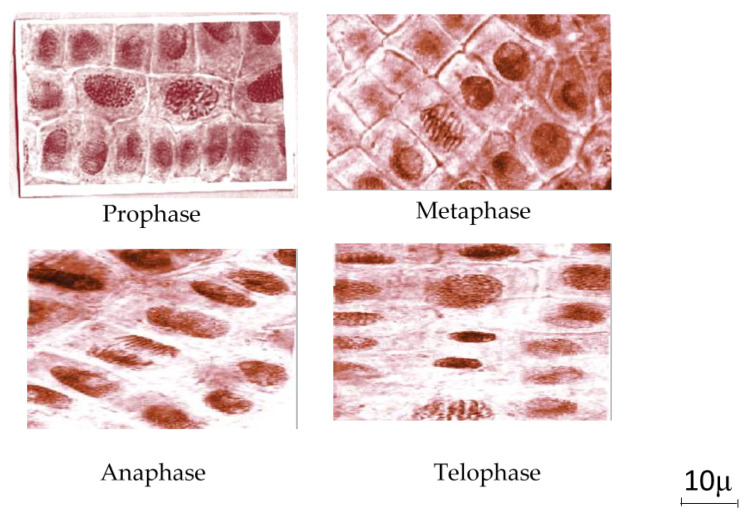
Microphotographs representing the stages of cell division observed in the control sample (normal mitotic phases: prophase, metaphase, anaphase, telophase).

Observations were made on the roots of wheat embryo stained with acetic orcein. Optical microscope readings were made every 24 h. The main changes to karyokinetic products are presented in Figure 6. In an observation of the changes we made, in a control sample compared to the normal mitotic division, there were phases whose layout is shown in Figure 7.

The microscopic examination of the roots of wheat germ in the case of the test solutions is highlighted: an inhibitory effect on cytokinesis induced by the most extractive aqueous solutions at high concentrations of 10% and 5%, and sometimes at low concentrations (0.66%); cell divisions as anaphase type, metaphases, telophase and cytokinesis, telophase and prophase with bridges, broken metaphases, most often for concentrations of 0.66%, and rare at concentrations of 10% and 5%; nuclei with aberrant forms, with elongated appearance, with 1–3 hypertrophied nucleolus and the presence of some cytotoxic effects.

#### 3.2.3. Evaluation of Acute Toxicity on Animals

**Figure 8:**
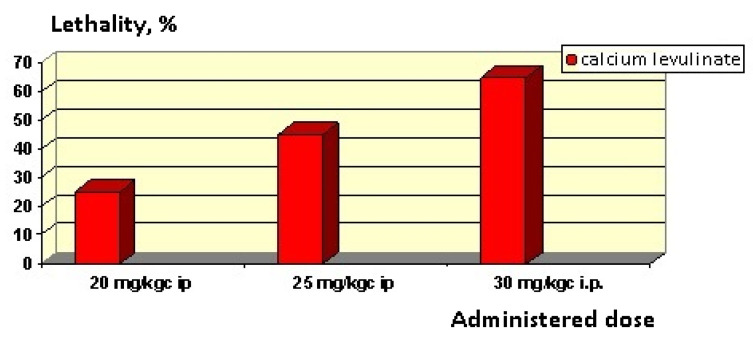
Lethality of calcium levulinate depending on the administered dose for mice.

For the value of the maximum tolerated dose for calcium levulinate in mice, it was determined to be 18.5 mg/kgc i.p. In Figure 8 we provide the corresponding lethality of the dose administered from active substances.

For calcium levulinate, the regression equation is: lethality (%) = 110.459 + 83.050 Logarithm_dose.

Based on the equation, it can be estimated that the dose corresponds to a value of 50% lethality. Accordingly:

50 = 110.459 + 83.050 Logarithm_LD50, 

and, logarithm_LD = (50–110.459)/96.050 = 1.4292,

LD 50 = 26.867 mg/Kgc i.p.

The value of the correlation coefficient R = 0.655 (higher than 0.5) indicates a bond, a fairly strong correlation between the dose and the lethal effect observed. Additionally, a coefficient of *p* < 0.05 indicates that the bond is very strong and significant.

##### Clinical Observations

At doses above the maximum tolerated one, the animals began to breathe heavily, as increasing the dose of the active substance movement is made more cumbersome and animals presented with hyperreactivity to tactile stimuli.

### 3.3. Mineral Composition of Mussel Shells

Table 5 presents the elemental concentration of the studied mussel shells, measured by ICP-MS analysis. The element concentration was expressed as parts per billion (ppb). 

There is a very large quantity of calcium in mussel shells (35,452.65 ppb), and there is also a large quantity of phosphorus (595.46 ppb). A moderate quantity of sodium and iron is present in mussel shells (Table 5). The results were also expressed in percentages (Table 6).

The results presented in Table 5 indicate calcium as the major constitutional component of the composition of mussel shells (95.88%).

### 3.4. Economic and Environmental Impact

By capitalizing the shells resulting from the industrial processing of mussels, calcium salts can be obtained for the pharmaceutical industry with low costs. The technological process of capitalizing on the waste resulting from the processing of mussels is efficient and non-polluting, simultaneously ensuring protection of the environment by reducing polluting materials.

At the same time, the use of waste in order to obtain new sources of calcium salts for the pharmaceutical industry has a significant social impact both in terms of creating new jobs and especially in terms of health, calcium being a macro-element essential for the health of the population.

In terms of the amount of waste resulting from the industrial processing of mussels, annually, farms specializing in growing seafood produce about 2.2 million tonnes of seafood. World mussel production continues to grow. People have used mussels as food for thousands of years. About 17 species are edible, of which the most commonly consumed are *Mytilus edulis*, *Mytilus galloprovincialis*, *Mytilus trossellus,* and *Perna canaliculus*. Mussels continued to be one of the cheapest seafoods in the market, with a value at around USD 2.00 per kg of live weight. Mussels are a low-fat, low-calorie food and an excellent source of proteins rich in essential amino acids, but also valuable mineral salts for the human body. The main producers of mussels are China, Chile, Spain, Thailand, and New Zealand. Mussels are some of the most famous delicacies. The beneficial properties of mussels have been appreciated by everyone. In Europe, where mussels have been cultivated for centuries, Spain has remained the industry leader [37,38].

In the current context generated by the COVID-19 pandemic, there is an increasing emphasis on adopting a healthy lifestyle that obviously includes a rational diet rich in valuable nutrients, an essential aspect for strengthening the immune system. As a result, seafood is one of the foods recommended by nutritionists to be consumed in increasing amounts. We can appreciate that in the future, the production of mussels will continue to register a continuous growth trend [38,39,40].

The correlation of the capitalization of mussel shells resulting as waste from mussel processing in order to obtain meat as raw material for the food industry, is one of the best solutions for the profitability of the process of integral processing of culture mussels and those fished naturally.

**Figure 9:**
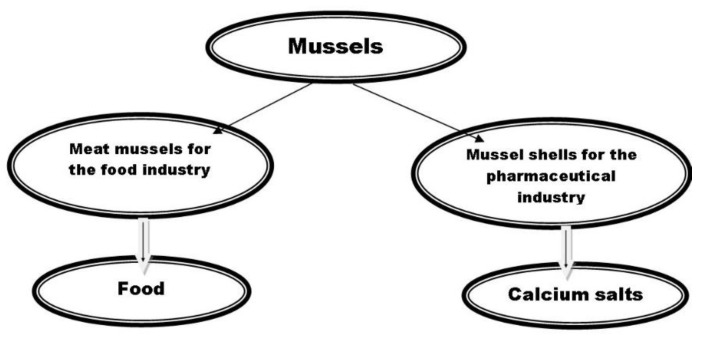
Integral technological processing of mussels.

The most important aspects related to the process of integral mussel processing are: abundance and therapeutic potential of the raw material and the resulting products, profitability, positive economic and social impact, and environmental protection through the development of non-polluting technologies (Figure 9).

## 4. Conclusions

The paper aimed to present original results concerning valuable waste (mussel shells) reutilization as secondary raw material to prepare pharmaceutically pure calcium salt, calcium levulinate. To recover the natural calcium existing in mussel shells, we used an original, patented technology. The newly prepared calcium salt was studied to verify whether it corresponds to use in the pharmaceutical industry. Our research results lead to the following conclusions: the technology used for extraction of calcium salts from mussel shells is simple, effective, and cheap. The prepared calcium salt (calcium levulinate) may be used in the pharmaceutical industry as meeting quality requirements provided by specific standards; elemental analysis of mussel shells showed low content of toxic metals; the toxicity of the calcium levulinate solutions on the roots of wheat germ at lower concentrations did not induce clear inhibition, which indicates low toxicity on plant cells; acute toxicity evaluated on experimental animals (*Swiss albino* mice) showed calcium levulinate to be of low toxicity; according to the analyses performed, calcium was noted as the majority constituent of mussel shells; the waste resulting from the industrial processing of mussel shells is a valuable natural source of calcium.

## Figures and Tables

**Table 1:** Preparation of test solutions.

Test Solutions *	V_Calcium levulinate solution_ (mL)	V_Distilled water_ (mL)	V_Total_ (mL)	Concentration (g% Calcium Levulinate)
A1	15.00	-	15.00	10.00%
A2	10.00	5.00	15.00	6.66%
A3	7.50	7.50	15.00	5.00%
A4	5.00	10.00	15.00	3.33%
A5	1.00	14.00	15.00	0.66%
A6	0.10	14.90	15.00	0.06%

* A represents test solutions of calcium levulinate with different concentrations.

**Table 2:** Characteristics of mussel shell calcium levulinate.

Characteristics	Limits Accepted by USP	Obtained Values
Melting point (°C)	119–125	122.5
pH solution 10%	7.8–8.5	7.9
Loss on drying 60 °C, 5 mmHg	10.5–12.0	10.8
Chloride content (%)	max. 0.070	0.052
Sulphate content (%)	max. 0.050	0.041
Reducing sugars	Absent	Absent
Identification		
Iodine/Iodinated Dinitrophenylhydrazine	CHI_3_ is formed Hydrazone is formed	CHI_3_ is formed Hydrazone is formed
Dosing with Na_2_EDTA	97.5–100.5%	98.73%

**Table 3:** Elemental analysis of the calcium levulinate.

	C%	H%	N%	S%
Assay values	44.04	5.37	0.34	0.14
Theoretical values	44.43	5.22	0.00	0.00

**Table 4:** The average results ± SD of the measurements of root elongation (mm) for calcium levulinate.

Time (Hours)	Control Sample (mm)	A1 (mm)	A2 (mm)	A3 (mm)	A4 (mm)	A5 (mm)	A6 (mm)
24	22.5 ± 0.45	11.5 ± 0.22	10.9 ± 0.93	12.5 ± 1.31	12.9 ± 0.26	13.4 ± 0.44	17.2 ± 0.48
48	42.7 ± 0.65	11.9 ± 0.82	12.6 ± 1.14	13.4 ± 1.06	13.5 ± 0.24	14.7 ± 0.42	24.2 ± 1.62
72	61.6 ± 0.55	12.6 ± 1.14	12.8 ± 1.71	14.5 ± 0.85	15.7 ± 0.62	15.4 ± 1.09	35.2 ± 1.02
96	84.2 ± 0.64	12.6 ± 0.78	12.8 ± 0.56	15. 2 ± 1.16	16.4 ± 1.24	17.2 ± 1.36	55.6 ± 0.78
120	105.3 ± 1.27	12.6 ± 0.71	12.8 ± 1.14	15.8 ± 1.76	16.9 ± 0.54	19.6 ± 1.18	86.4 ± 1.08
E%/120 h	-	−88.03	−87.84	−84.99	−83.95	−81.38	−17.94

where A = test solutions of calcium levulinate with different concentrations; Control sample = blank solution.

**Table 5:** Elemental concentration of mussel shells in parts per billion (ppb).

Element	Concentration, ppb
Ca	35,452.65 ± 1883.07
P	595.46 ± 16.73
Mg	53.22 ± 2.60 *
Na	467.21 ± 2.39 *
Fe	255.38 ± 0.60 **
Zn	30.14 ± 0.13 **
Mn	27.07 ± 0.66 **
Cr	93.20 ± 1.62 *
Pb	0.85 ± 1.12 *
Cd	BDL
Hg	BDL

* *p* ˂ 0.01; ** *p* ˂ 0.001; BDL = below detection limits.

**Table 6:** Elemental concentration of mussel shells in percentages.

Element	Concentration, %
Calcium	95.88 ± 1.2 *
Phosphorus	1.61 ± 0.2 *
Magnesium	0.14 ± 0.04 **
Natrium	1.26 ± 0.3 *
Iron	0.69 ± 0.02 **
Others	0.42 ± 0.03 **

* *p* ˂ 0.01; ** *p* ˂ 0.001.

## Data Availability

There are no data available for this publication.

## References

[1] Costa S., Afonso C., Bandarra N.M., Gueifao S., Castanheira I., Carvalho M.L., Cardoso C., Nunes M.L. (2013). The emerging farmed fish species meagre (*Argyrosomus regius*): How culinary treatment affects nutrients and contaminants concentration and associated benefit-risk balance. Food Chem. Toxicol..

[2] Rittenschober D., Stadlmayr B., Nowak V., Du J., Charrondiere U.R. (2016). Report on the development of the FAO/INFOODS user database for fish and shellfish (uFiSh)—Challanges and possible solutions. Food Chem..

[3] Carrington E., Waite J.H., Sarà G., Sebens K.P. (2015). Mussels as a model system for integrative echomecanics. Ann. Rev. Mar. Sci..

[4] Galimany E., Wikfors G.H., Dixon M.S., Newell C.R., Meseck S.L., Henning D., Li Y.Q., Rose J.M. (2017). Cultivation of the Ribbed mussel (*Geukensia demissa*) for nutrient bioextraction in an urban estuary. Environ. Sci. Technol..

[5] Mititelu M., Moroşan E., Neacșu S.M., Ioniţă E.I. (2018). Research regading the pollution degree from Romanian Black Sea coast. Farmacia.

[6] Newell R.I.E.J. (2004). Ecosystem influences of natural and cultivated populations of suspension-feeding bivalve molluscs: A review. Shellfish Res..

[7] Çelik M.Y., Karayücel S., Karayücel I., Öztürk R., Eyüboglu B.J. (2012). Meat yield, condition index, and biochemical composition of mussels (*Mytilus galloprovincialis Lamarck*, 1819) in Sinop, south of the Black Sea. Aquat. Food Prod. Technol..

[8] Grienke U., Silke J., Tasdemir D. (2014). Bioactive compounds from marine mussels and their effects on human health. Food Chem..

[9] Holmer M., Thorsen S.W., Carlsson M.S., Kjerulf P.J. (2015). Pelagic and benthic nutrient regeneration processes in mussels cultures (*Mytilus edulis*) in a Eutrophic Coastal Area (Skive Fjord, Denmark). Estuaries Coasts.

[10] Fuentes A., Fernández-Segovia I., Escriche I., Serra J.A. (2009). Comparison of physico-chemical parameters and composition of mussels (*Mytilus galloprovincialis* Lmk.) from different Spanish origins. Food Chem..

[11] Freites L., Fernández-Reiriz M.J., Labarta U. (2002). Fatty acid profiles of *Mytilus galloprovincialis* (Lmk) mussels of subtidal and rocky shore origin. Comp. Biochem. Physiol. Part B Biochem. Mol. Biol..

[12] Murphy J.K., Moone D.B., Mann J.N., Nichols D.P., Sinclair J.A. (2002). Lipid, FA, and sterol composition of New Zealand green lipped mussel (*Perna canaliculus*) and Tasmanian blue mussel (*Mytilus edulis*). Lipids.

[13] Di Nunzio M., Valli V., Bordoni A. (2011). Pro- and anti-oxidant effect of polyunsaturated fatty acid supplementation in HepG2 cells. Prostaglandins Leukot. Essent. Fat. Acids.

[14] Kesavulu M.M., Kameswararao B., Apparao C., Kumar E.G., Harinarayan C.V. (2002). Effect of omega-3 fatty acids on lipid peroxidation and antioxidant enzyme status type 2 diabetic patients. Diabetes Metab..

[15] Saravanan P., Davidson N.C., Schmidt E.B., Calder P.C. (2010). Cardiovascular effects of marine omega-3 fatty acids. Lancet.

[16] Hamester M.R.R., Balzer P.S., Becker D. (2012). Characterization of calcium carbonate obtained from oyster and mussel shells and incorporation in polypropylene. Mater. Res..

[17] Ituen E.U. (2015). Mechanical and chemical properties of selected molluse shells in Nigeria. Int. J. Agric. Policy Res..

[18] Morris J.P., Thierry B., Gauthier C. (2019). Shells from aquaculture: A valuable biomaterial, not a nuisance waste product. Rev. Aquac..

[19] Mihele D., Mititelu M. (2010). Process for obtaining calcium levulinate from mussels shells, involves deproteinisation, treatment with levulinic acid and purification of shell powder deproteinisatio.

[20] Onoda H., Nakanishi H., Takenaka A.J. (2012). Preparation of calcium phosphates with *Corbicula Shells*. J. Ecotechnol. Res..

[21] Mititelu M., Ioniţă A.C., Moroşan E. (2014). Research regarding integral processing of mussels from Black Sea. Farmacia.

[22] (2003). The National Formulary XVIII.

[23] Gordon B., Kough O.S., Proskouriakoff A.J. (1933). Studies on calcium levulinate with special reference to the influence on edema. Lab. Clin. Med..

[24] Proskouriakoff A. (1933). Some salts of levulinic acid. JACS.

[25] Sharath B.O., Tiwari R., Mal S.S., Dutta S. (2019). Straightforward synthesis of calcium levulinate from biomass-derived levulinic acid and calcium carbonate in egg-shells. Mater. Today Proc..

[26] Nuţă D., Dinu M. (2005). Testarea actiunii fitobiologice a unor noi N-(2-dialchilaminoacetil)-benzanilide divers substituite. Farmacia.

[27] AVMA (2013). Guidelines for the Euthanasia of Animals.

[28] OECD (2001). Guidelines for Testing of Chemicals. Acute Oral Toxicities Up and Down Procedure.

[29] Ahmed M. (2015). Acute toxicity (lethal dose 50 calculation) of herbal drug Somina in rats and mice. Pharmacol. Pharm..

[30] Gadaleta D., Vuković K., Toma C., Lavado G.J., Karmaus A.L., Mansouri K., Kleinstreuer N.C., Benfenati E., Roncaglioni A. (2019). SAR and QSAR modeling of a large collection of LD50 rat acute oral toxicity data. J. Cheminform..

[31] Raj J., Chandra M., Dogra T.D., Pahuja M., Raina A. (2013). Determination of median lethal dose of combination of endosulfan and cypermethrin in wistar rat. Toxicol. Int..

[32] AOAC (1990). Association of Official Analytical Chemists, Official Methods of Analysis.

[33] Al-Obaidi F.A., Mehdi B.I., Al- Shadeedi S.M. (2012). Identification of inorganic elements in egg shell of some wild birds in Baghdad. Adv. Appl. Sci. Res..

[34] Chang F., Li G., Haws M., Niu T. (2007). Element concentrations in shell of Pinctada margaritifera from French Polynesia and evaluation for using as a food supplement. Food Chem..

[35] Mair P., Wilcox R. (2020). Robust statistical methods in R using the WRS2 package. Behav. Res. Meth..

[36] Bojiţă M., Roman L., Săndulescu R., Oprean R. (2003). Analiza şi Controlul Medicamentelor. Metode Instrumentale în Analiza şi Controlul Medicamentelor.

[37] FAO (2018). The State of World Fisheries and Aquaculture—Meeting the SUSTAINABLE Development Goals. http://www.fao.org/3/i9540en/i9540en.pdf.

[38] Venugopal V., Gopakumar K. (2017). Shellfish: Nutritive value, health benefits, and consumer safety. Compr. Rev. Food Sci. Food Saf..

[39] Jacquet J., Sebo J., Elder M. (2017). Seafood in the future: Bivalves are better. Solutions.

[40] Suplicy F.M. (2018). A review of the multiple benefits of mussel farming. Rev. Aquac..

